# Microfluidic-Based Biosensor for Sequential Measurement of Blood Pressure and RBC Aggregation Over Continuously Varying Blood Flows

**DOI:** 10.3390/mi10090577

**Published:** 2019-08-30

**Authors:** Yang Jun Kang

**Affiliations:** Department of Mechanical Engineering, Chosun University, 309 Pilmun-daero, Dong-gu, Gwangju 61452, Korea; yjkang2011@chosun.ac.kr; Tel.: +82-62-230-7052; Fax: +82-62-230-7055

**Keywords:** RBCs aggregation, blood pressure, ACS (air-compressed syringe), microfluidic device, continuous varying flow rates

## Abstract

Aggregation of red blood cells (RBCs) varies substantially depending on changes of several factors such as hematocrit, membrane deformability, and plasma proteins. Among these factors, hematocrit has a strong influence on the aggregation of RBCs. Thus, while measuring RBCs aggregation, it is necessary to monitor hematocrit or, additionally, the effect of hematocrit (i.e., blood viscosity or pressure). In this study, the sequential measurement method of pressure and RBC aggregation is proposed by quantifying blood flow (i.e., velocity and image intensity) through a microfluidic device, in which an air-compressed syringe (ACS) is used to control the sample injection. The microfluidic device used is composed of two channels (pressure channel (PC), and blood channel (BC)), an inlet, and an outlet. A single ACS (i.e., air suction = 0.4 mL, blood suction = 0.4 mL, and air compression = 0.3 mL) is employed to supply blood into the microfluidic channel. At an initial time (*t* < 10 s), the pressure index (*PI*) is evaluated by analyzing the intensity of microscopy images of blood samples collected inside PC. During blood delivery with ACS, shear rates of blood flows vary continuously over time. After a certain amount of time has elapsed (*t* > 30 s), two RBC aggregation indices (i.e., *S_EAI_*: without information on shear rate, and erythrocyte aggregation index (*EAI*): with information on shear rate) are quantified by analyzing the image intensity and velocity field of blood flow in BC. According to experimental results, *PI* depends significantly on the characteristics of the blood samples (i.e., hematocrit or base solutions) and can be used effectively as an alternative to blood viscosity. In addition, *S_EAI_* and *EAI* also depend significantly on the degree of RBC aggregation. In conclusion, on the basis of three indices (two RBC aggregation indices and pressure index), the proposed method is capable of measuring RBCs aggregation consistently using a microfluidic device.

## 1. Introduction

Several factors including smoking, pressure, and glucose contribute to increasing the risk of cardiovascular diseases (CVDs) including coronary heart disease, myocardial infarction, and atherosclerosis. CVDs occur without any symptoms, and cause serious complications or unexpected deaths [1]. For this reason, they have been considered as the top cause of deaths globally. Impaired microcirculations including clotting or interrupted blood flows lead to symptoms associated with organ failure or mortality. Currently, CVDs have been diagnosed with biochemical parameters such as biomarkers [2,3], cholesterol, and glucose. However, the biochemical analyses do not provide sufficient information on the early detection of impaired microcirculations. As the strong association between coronary heart diseases and hemorheological properties was reported [4], the rheological properties have been employed to detect pathophysiological processes of vascular diseases or clinical states [5]. Because the number of RBCs (red blood cells) is significantly higher than the other cells (platelet, white blood cell), hemorheological properties of blood are dominantly determined by those of the RBCs [5,6]. Hemorheological properties including haematocrit [7,8,9,10], blood viscosity [11,12,13,14], RBC aggregation [15,16,17,18,19,20,21,22], and RBC deformability [14,23,24,25,26,27,28,29,30,31,32,33,34,35,36,37,38] have been proposed for the early detection of CVDs [39,40]. RBC aggregation leads to altering the hemorheological property, especially in lower blood flows of post-capillary venules [5,41]. RBCs aggregates and forms rouleaux at stasis or under low shear rates, which causes the hemorheological property to vary [42]. However, RBC aggregates are completely dispersed at higher shear rates. Hematological diseases including malaria [43], diabetes [44,45], and hypertension [46] contribute significantly to enhancing RBC aggregation. RBC aggregation is determined by several factors such as plasma proteins, membrane deformability, and hematocrit [47]. Among them, hematocrit has a significant influence on RBC aggregation. For this reason, to measure RBC aggregation effectively, hematocrit or the associated blood property (i.e., blood viscosity, or blood pressure) should be monitored simultaneously. Furthermore, as RBC aggregation is varied depending on blood flow conditions, it should be measured over shear rate.

Because a microfluidic-based technique can provide several advantages such as small volume requirement, disposable device, and short measurement time, RBCs aggregation of blood has been quantified under microfluidic platform [48,49,50]. After a microfluidic channel is filled with blood, blood flows or stops by sequentially operating external devices such as a syringe pump [51,52], pinch valve [47], and vacuum pump [49]. When blood flows decrease to extremely lower shear rates, RBCs tend to aggregate over time. Several measurement quantifies such as photometric intensity [49,50], electric conductivity [53,54], optical tweezer [16], and microscopic image intensity [18,52,55,56,57] have been adopted to obtain a syllectogram (i.e., quantity versus time) [54]. Thereafter, the RBCs aggregation index is calculated from syllectogram. On the basis of microscopic image of individual RBCs flowing in the microfluidic channel, it is reported that fibrinogen or polymer dextran contribute to enhancing RBCs aggregation, and stabilizing RBC clusters in microcapillary flows [58,59]. Aggregate sizes obtained by the image processing technique are used to quantify spatial distributions of RBCs aggregated in a T-shaped bifurcation [60]. A linear viscoelasticity property is suggested to evaluate the effect of hematocrit or dextran on RBCs aggregation [61]. 

Although several methods are reported to measure RBCs aggregation under a microfluidic platform, there are still several limitations on RBCs aggregation measurements under microfluidic environments. First, to aggregate or disaggregate RBCs in a microfluidic channel, blood flows or stops periodically by using external bulky facilities, including a syringe pump, vacuum pump, and solenoid valve. Owing to the bulky and expensive facility, most of the methods are demonstrated in a well-equipped laboratory. To be used in resource-limited environments, the bulky facility should be replaced with a simple delivery method. Second, RBCs aggregation varies considerably depending on changes of several factors, such as hematocrit, plasma proteins, and RBC membrane fragility. For this reason, while measuring RBCs aggregation, it is simultaneously required to monitor the contribution of hematocrit or base solution of the blood sample, as summarized in Table 1. Although blood viscosity has been used to effectively monitor the contribution of hematocrit, the flow rate of each fluid should be kept constant using external syringe pumps. To remove syringe pumps, blood viscosity should be replaced with the other biophysical property of blood (i.e., blood pressure). 

In this study, to resolve these issues, first, a simple blood delivery method (i.e., air-compressed syringe (ACS)) is adopted to supply blood samples into a microfluidic device. During blood delivery from ACS to the microfluidic device, the air cavity tends to increase inside ACS, which results in decreasing pressure inside ACS as time elapses. Then, the blood flow rate tends to decrease in a microfluidic channel. Under unique features of ACS, RBCs aggregation occurs when the blood flow rate decreases to a corresponding value of lower shear rate. Because RBCs aggregation tends to vary depending on shear rate (or blood flow rate), variations of RBC aggregation should be monitored under various shear rates. Thus, the contribution of shear rate is evaluated by obtaining RBC aggregation at stasis and RBCs aggregation under varying flow rates, simultaneously. Two RBCs aggregation indices (i.e., *S_EAI_*: without information on shear rate, and erythrocyte aggregation index (*EAI*): with information on shear rate) are quantified by analyzing image intensity and velocity fields of blood flows. Here, the blood flow rate is quantified using micro particle image velocimetry. The shear rate of blood flow is then estimated using the analytic formula of a rectangular channel with a low aspect ratio. For this reason, the ACS contributes to removing on–off flow control, which is considered as a necessary step in the previous method [62]. Second, the blood pressure index (*PI*) is employed to quantify contribution of hematocrit in an individual blood sample as an alternative to blood viscosity. Compared with the previous study [62], the compliance effect of the tube decreases substantially by increasing the thickness of the tube from 250 μm to 500 μm. Owing to lower compliance of the tube (i.e., high stiffness), the proposed method does not require to stop blood flow using a pinch valve. To verify the relationship between *PI* and blood viscosity, *PI* and blood viscosity are simultaneously measured and quantified for several blood samples.

## 2. Materials and Methods 

### 2.1. Blood Sample Preparation

The protocol was approved by the Ethics Committee of Chosun University Hospital (CUH) (CHOSUN 2018-05-11). All experiments were performed by ensuring that the procedures involved were appropriate and humane. Concentrated RBCs and FFP (fresh frozen plasma) were purchased from the Gwangju–Chonnam blood bank (Gwangju, Korea). Concentrated RBCs were stored in citrate phosphate dextrose adenine (CDPA-1) anticoagulant solution. They were stored at 4 °C and −25 °C, respectively. To remove the anticoagulant solution from concentrated RBCs, concentrated RBCs (~7 mL) and PBS (phosphate-buffered saline) (1×, pH 7.4, Gibco, Life Technologies, New York, NY, USA) were added and mixed in a 15 mL tube. When installing the tube in a centrifuge (Allegra X-30R benchtop, Beckman Coulter, Brea, CA, USA) and setting 4000 rpm for 10 min, liquid (upper layer) and cells (lower layer) due to differences in density were completely separated in the tube. Then, normal RBCs as a lower layer were collected by removing a buffy layer and PBS as an upper layer [36]. The washing procedure described above was repeated twice. FFP was melted at room temperature. Blood samples were prepared by adding RBCs into specific base solutions (i.e., PBS solution, plasma, and dextran solution). Blood samples were then kept at 4 °C before the blood test. In this study, instead of whole blood collected from the volunteer, the concentrated RBCs and plasma purchased from the blood bank were employed to appropriately control the degree of RBCs aggregation and RBCs’ deformability. The blood sample named in this study could be regarded as RBCs suspended in a specific solution (i.e., RBCs suspended in plasma, or RBCs suspended in PBS). First, to evaluate the effect of the haematocrit and base solution on pressure and RBC aggregation, the haematocrit (*Hct* = 30%, 40%, and 50%) was prepared by adding normal RBCs into PBS solution, plasma, and dextran solution (*C_dextran_* = 10 mg/mL). Second, to stimulate RBCs aggregation of the blood sample, four different concentrations of dextran solution (*C_dextran_* = 5, 10, 15, and 20 mg/mL) were diluted by mixing dextran (*Leuconostoc* spp., *M_W_* = 450–650 kDa, Sigma-Aldrich, St. Louis, MO, USA) with PBS solution. Subsequently, blood samples (*Hct* = 50%) were prepared by adding normal RBCs to specific concentrations of dextran solution. Third, to vary RBC deformability of blood sample, three different concentrations of glutaraldehyde (GA) solution (*C_GA_* = 5, 10, and 15 µL/mL) were diluted by mixing GA solution (grade II, 25% in H_2_O, Sigma-Aldrich, St. Louis, MO, USA) into PBS solution. Normal RBCs were then hardened after dipping normal RBCs into each concentration of GA solution for 10 min. Hardened blood samples (*Hct* = 50%) were then prepared by adding hardened RBCs into the PBS solution. 

### 2.2. Fabrication of a Microfluidic Device and Experimental Procedure

**Figure 1:**
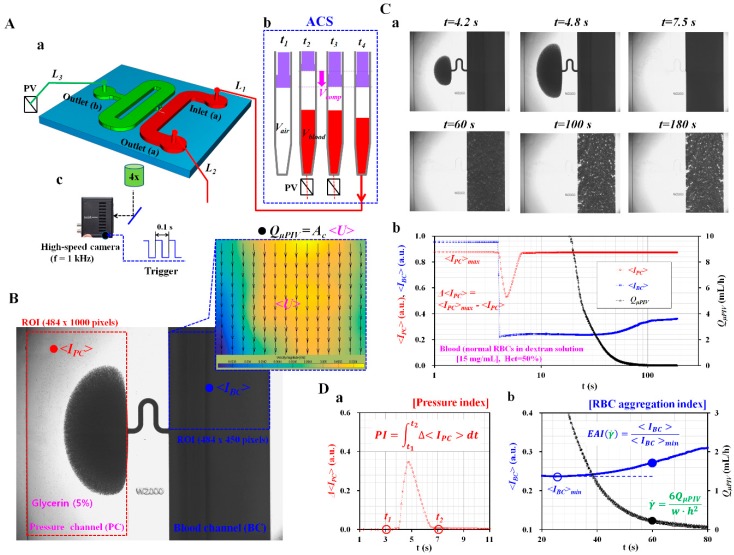
A proposed method for sequential measurement of blood pressure and red blood cells’ (RBCs) aggregation over varying blood flows. (**A**) A schematic diagram of the proposed method, including disposable air-compressed syringe (ACS), a microfluidic device, two pinch valves (PVs), and an image acquisition system. (**a**) A microfluidic device was composed of an inlet (a), two outlets (a,b), and two channels (pressure channel (PC), and blood channel (BC)). (**b**) The ACS was operated in four processes: air suction (V_air_) at *t* = *t*_1_, blood suction (*V_blood_*) at *t* = *t*_2_, air compression (*V_comp_*) by clamping outlet tube (L2) with pinch valve at *t* = *t*_3_, and blood delivery by removing PV (*t* = *t*_4_). (**c**) A high-speed camera with a frame rate of 1 kHz was employed to capture two sequential microscopic images at an interval of 0.1 s. (**B**) Two regions of interest (ROI) for evaluating three parameters (*<I_PC_>*, *<I_BC_>*, and *Q_μPIV_*). (**C**) As a preliminary demonstration, the blood sample was supplied into a microfluidic device from the ACS. (**a**) Sequential microscopic images captured at a specific time (*t*) (*t* = 4.2 s, 4.8 s, 7.5 s, 60 s, 100 s, and 180 s). (**b**) Temporal variations of three parameters (*<I_PC_>*, *<I_BC_*>, and *Q_μPIV_*) over time. (**D**) Quantification of pressure and RBC aggregation. (**a**) Blood pressure was quantified as pressure index (*PI*) obtained by integrating Δ*<I_PC_>* from *t* = *t*_1_ to *t* = *t*_2_. (**b**) Variations of RBCs aggregation were obtained as erythrocyte aggregation index (*EAI*) over shear rate (i.e., *EAI* vs. $\dot{\gamma }$).

A microfluidic device for measuring pressure and RBC aggregation sequentially consisted of two channels (pressure channel [PC], and blood channel [BC]), an inlet (a), and two outlets (a,b), as shown in Figure 1(A-a). The inlet of the PC (width = 2000 μm, and length = 40 mm) was connected to the middle position of the BC (width = 2000 μm, and length = 22 mm) with a serpentine channel (width = 100 μm, and length = 1750 μm). The channel depth of the microfluidic device was fixed at 100 μm. Conventional micro-electromechanical-system fabrication techniques, including photolithography and deep silicon etching (i.e., Deep RIE [Reactive Ion Etching]) were employed to fabricate a silicon-master mold (i.e., 4 inch silicon wafer). Polydimethylsiloxane (PDMS) (Sylgard 184, Dow Corning, Midland, MI, USA) was mixed with a curing agent at a ratio of 10:1. After the silicon master mold was placed in a petri dish (150 × 20 mm^2^, crystal grade polystyrene, SPL Lifescience Co., Gyeonggi-Do, Korea), the PDMS mixture was poured on the master mold. Air bubble in PDMS was removed with a vacuum pump (WOB-L Pump, Welch, Gardner Denver, Milwaukee, WI, USA) for 1 h. After curing the PDMS mixture in a convective oven at 70 °C for 1 h, a PDMS block was peeled off from the mold, and cut with a razor blade. Three ports were punched with a biopsy punch (outer diameter = 1.2 mm). After surfaces of the PDMS block and a slide glass were treated with an oxygen plasma system (CUTE-MPR, Femto Science Co., Gyeonggi-Do, Korea), a microfluidic device was finally prepared by bonding the PDMS block on the slide glass.

As shown in Figure 1(A-a), two polyethylene tubes corresponding to *L*_1_ (length = 300 mm, inner diameter = 500 μm, and thickness = 500 μm) and *L*_2_ (length = 200 mm, inner diameter = 500 μm, and thickness = 500 μm) were tightly fitted to inlet (a) and outlet (a), respectively. The end of the tube (*L*_1_) was connected to the syringe needle. Before compressing the air cavity inside the ACS, the tube (*L*_1_) connected to the outlet of the ACS was clamped using a pinch valve. Additionally, one tube corresponding to *L*_3_ (length = 200 mm, inner diameter = 500 μm, and thickness = 500 μm) was fitted to the outlet (b) of the PC. The end of the tube (*L*_3_) was clamped with a pinch valve (PV). When compared with the previous study [62], the thickness of the tube increased from 250 μm to 500 μm. The compliance effect of the tube decreased substantially. Because of lower compliance (or higher stiffness) of the tube, the method does not require to stop blood flows using a pinch valve as a critical step.

To remove the air bubble in the channels and avoid non-specific binding of plasma proteins to the inner surface of the channels, all channels were filled with BSA (bovine serum albumin) solution (*C_BSA_* = 2 mg/mL) through the outlet (b) with a disposable syringe. After 5 min had elapsed, all channels were newly filled with Glycerin solution (*C_Glycerine_* = 5%) with a disposable syringe. Thereafter, the end of the tube (*L*_3_) connected to the outlet (b) of the PC was completely clamped with the PV.

As shown in Figure 1(A-b), an air-compressed syringe (ACS) consisted of a disposable syringe (~1 mL) and a pinch valve (PV). The ACS was composed of four operational processes: air suction (*V_air_*) at *t* = *t*_1_, blood suction (*V_blood_*) at *t* = *t*_2_, air compression (*V_comp_*) by clamping the tube (*L*_2_) with PV at *t* = *t*_3_, and blood delivery by releasing the PV (*t* = *t*_4_). In other words, first, the air cavity was set to *V_air_* by moving the plunger backward. Second, blood was sucked to V_blood_ by moving the plunger backward. Third, before compressing the air cavity inside ACS, a PV was installed near the syringe needle, especially to stop the blood flow from ACS to a microfluidic device. Then, the air cavity was compressed about *V_comp_* by moving the plunger forward. Here, a micro manipulator was employed to accurately adjust the air cavity. At last, blood was then supplied from the ACS to a microfluidic channel by removing the PV.

The microfluidic device was positioned on an optical microscope (BX51, Olympus, Tokyo, Japan) equipped with a 4× objective lens (NA = 0.1). As shown in Figure 1(A-c), a high-speed camera (FASTCAM MINI, Photron, Tokyo, Japan) was employed to capture microscopic images of blood flows in microfluidic channels. The camera offered a spatial resolution of 1280 × 1000 pixels. Each pixel corresponded to 10 µm. With a function generator (WF1944B, NF Corporation, Yokohama, Japan), a pulse signal with period of 0.1 s triggered the high-speed camera. Microscopic images were sequentially captured at a frame rate of 1 kHz and at an interval of 0.1 s. All experiments were conducted at a room temperature of 25 °C.

### 2.3. Quantification of Image Intensity and Blood Flow-Rate

Pressure and RBC aggregation were sequentially obtained by quantifying image intensity of blood flows in PC and BC, respectively. As shown in Figure 1B, to evaluate pressure, a region of interest (ROI, 484 × 1000 pixels) was selected within the PC. As a measurement principle of pressure, pressure was measured by analyzing blood volume collected in the PC. At a higher pressure, blood flowed into the PC from the BC. However, at a sufficient low pressure, blood flowed from PC to BC reversely. As reported in a previous study [57], the amount of RBCs stacked in PC caused the deterioration of the measurement accuracy of pressure. Thus, instead of PBS, a higher density of glycerin solution as counter fluid was initially filled to float RBCs in PC, which intended to minimize RBCs stacked in the PC. Here, the glycerin solution as counter fluid in PC was diluted with 1× PBS. Because glycerin solution and PBS solution have the same osmotic pressure, there was no problem related to RBCs’ lysis in PC through all experiments. As blood volume collected in PC was linearly proportional to pressure [57], variation of pressure was obtained by analyzing image intensity of blood in the PC. Then, the image intensity of RBCs collected in the PC (*<I_PC_>*) was obtained by conducting digital image processing with a commercial software package (Matlab 2019, Mathworks, Natick, MA, USA). Additionally, to quantify RBCs aggregation of blood, averaged image intensity of blood flows in BC (*<I_BC_>*) was obtained by analyzing image intensity of RBCs distributed within a ROI (484 × 450 pixels). To quantify blood flow rate supplied from the ACS, the velocity field of blood flows in BC was obtained by conducting a time-resolved micro-PIV (particle image velocimetry) technique. The size of the interrogation window was 64 × 64 pixels. The window overlap was 50%. The obtained velocity field was validated with a median filter. According to the formula of DOC (depth of correlation) [64,65], DOC was estimated as 324.8 µm. Because DOC is sufficiently higher than channel depth (i.e., *h* = 100 µm), it is reasonably estimated that all RBCs can contribute to calculating the velocity field in the ROI of the microfluidic channel. Thus, the micro-PIV technique can measure averaged velocity field through depth direction. The averaged velocity (*<U>*) was calculated as an arithmetic average over the ROI. Subsequently, the flow rate of blood flows in BC (i.e., *Q_µPIV_*) was estimated by multiplying the averaged velocity (*<U>*) by the cross-sectional area with a rectangular channel (*A_c_*) (i.e., *Q_µPIV_* = *<U>*∙*A_c_*).

As shown in Figure 1C, as a preliminary study, blood sample (*Hct* = 50%) was prepared by adding normal RBCs to a specific concentration of dextran solution (i.e., *C_dextran_* = 15 mg/mL). Figure 1(C-a) showed the corresponding microscopic images captured at a specific time (*t* = 4.2 s, 4.8 s, 7.5 s, 60 s, 100 s, and 180 s). Figure 1(C-b) depicted temporal variations of image intensity (*<I_PC_>*, and *<I_BC_>*) and blood flow rate (*Q_μPIV_*) for 180 s. At an initial time (*t* < 10 s), RBCs were collected and removed in PC depending on pressure in BC. By conducting digital imaging processing, *<I_PC_>* showed a bell-shaped function with respect to time. The variation of *<I_PC_>* was calculated as Δ*<I_PC_>* = *<I_PC_>_max_* − *<I_PC_>*. As shown in Figure 1(D-a), pressure index (*PI*) was newly suggested as $PI=\int_{t=t_{1}}^{t=t_{2}}\Delta \langle I_{PC}\rangle dt$. Because the blood sample was continuously supplied into the microfluidic device from CS, the air cavity inside the ACS increased over time. On the basis of ideal gas law under isothermal condition (i.e., *P* × *V* = constant, *P*: pressure, and *V*: air cavity), the increase in the air cavity led to a decrease in pressure inside the ACS. For this reason, blood flow rate decreased gradually in a microfluidic channel. During blood delivery with ACS, shear rates of blood flows decreased over time. After a certain amount of time had elapsed (*t* > 30 s), the blood flow rate decreased to a lower flow rate, which resulted in inducing RBC aggregation substantially. RBCs were aggregated continuously. As a result, RBCs aggregation contributed to increasing *<I_BC_>* over time. By referring to previous studies [51,55,56], the RBC aggregation index with respect to shear rate was newly suggested as *EAI* (erythrocyte aggregation index) ($\dot{\gamma }$) by dividing *<I_BC_>* obtained at a specific shear rate with the minimum value of *<I_BC_>* (i.e., *EAI* ($\dot{\gamma }$) = <*I_BC_* ($\dot{\gamma }$)>/*<I_BC_>_min_*). The characteristic shear rate for each flow rate was calculated as $\dot{\gamma }=\frac{6Q_{\mu PIV}}{w\;h^{2}}$ for a rectangular channel with lower aspect ratio (width = *w*, and depth = *h*) [56]. On the other hand, as represented in Figure 3(B-b), without obtaining information on the blood flow rate, the RBC aggregation index was suggested as *S_EAI_* by averaging Δ*<I_BC_>* from *t* = *t*_1_ to *t* = *t*_2_ (i.e., $S_{EAI}=\frac{1}{\left(t_{2}-t_{1}\right)}\int_{t=t_{1}}^{t=t_{2}}\Delta \langle I_{BC}\rangle dt$). Here, t_1_ denotes the initial time, where *<I_BC_>* exhibits a minimum value. *t*_2_ means final time of each experiment. Δ*<I_BC_>* denotes as Δ*<I_BC_>* = *<I_BC_>* − *<I_BC_>_min_*. Thus, in this study, RBC aggregation of each blood sample was evaluated using two indices, that is, *EAI* (i.e., with information on shear rate) and *S_EAI_* (i.e., without information on shear rate).

### 2.4. Statistical Analysis

Statistical significance was obtained by conducting statistical analysis with a commercial software (SPSS Statistics version 22, IBM Corp., Armonk, NY, USA). Analysis of variance (ANOVA) test was used to verify significant difference between comparative groups. In addition, linear and non-linear regression analyses were conducted to verify a significant relationship between two parameters. If *p*-value was less than 0.05, the result exhibited a significant difference within a 95% confidence interval. 

## 3. Results and Discussion

### 3.1. Effect of Air Cavity Adjusted inside the ACS on Pressure Index (PI)

**Figure 2:**
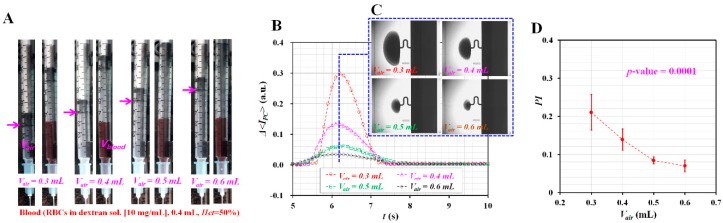
Quantitative evaluations of the effect of the air cavity adjusted inside the ACS on pressure index (*PI*). Here, blood sample (normal RBCs in dextran solution [10 mg/mL], *Hct* = 50%, and blood volume = 0.4 mL) were sucked into the ACS. The air cavity was compressed about *V_comp_* = 0.3 mL consistently. (**A**) Snapshots obtained at varying air cavity (*V_air_* = 0.3, 0.4, 0.5, and 0.6 mL). (**B**) Temporal variations of Δ*<I_PC_>* with respect to *V_air_*. (**C**) Microscopic images captured at a specific time when Δ*<I_PC_>* had the highest value depending on *V_air_*. (**D**) Variations of pressure index (*PI*) with respect to *V_air_*.

Pressure difference (i.e., Δ*P* = *P_ACS_* − *P_atm_*) between atmosphere pressure (*P_atm_*) and pressure inside ACS (*P_ACS_*) was acted as an external force, which caused the supply of bloods from ACS to a microfluidic device. According to a well-known Hagen–Poiseuille relation (i.e., Δ*P* = *R_f_* × *Q*, *R_f_*: fluidic resistance, and *Q*: flow rate), the higher the pressure inside the ACS, the faster blood flows. According to ideal-gas law under isothermal condition (i.e., pressure [*P*] × volume [*V*] = constant), the pressure difference was estimated. First, before compressing the air cavity inside ACS (*V_air_*), pressure and volume were given as *P*_1_ = *P_atm_* and *V*_1_ = *V_air_*, respectively. Second, when compressing air cavity to *V_comp_*, air cavity decreased to *V*_2_ = *V_air_* − *V_comp_*. On the basis of ideal gas law, *P*_2_ = *P_ACS_* was estimated as *P_ACS_* = *P*_1_ × *V*_1_/*V*_2_ = *P_atm_* × *V_air_*/(*V_air_* − *V_comp_*). Thus, Δ*P* = *P_ACS_* − *P_atm_* was derived as Δ*P* = *P_atm_* × *V_comp_*/(*V_air_* − *V_comp_*). In other words, when *V_comp_* was fixed at *V_comp_* = 0.3 mL, ΔP can be changed by adjusting the air cavity (*V_air_*) inside ACS. For this reason, it is required to evaluate the effect of the air cavity (*V_air_*) inside the ACS on variation of pressure in the microfluidic channel (i.e., BC). Variations of *<I_PC_>* and pressure index (*PI*) were evaluated by appropriately adjusting the air cavity (*V_air_*). Here, the blood sample (*Hct* = 50%) was prepared by adding normal RBCs into a specific concentration of dextran solution (i.e., *C_dextran_* = 10 mg/mL). As depicted in Figure 2A, the air cavity (*V_air_*) was adjusted to *V_air_* = 0.3 mL, 0.4 mL, 0.5 mL, and 0.6 mL. For convenience, *V_blood_* and *V_comp_* were fixed as *V_blood_* = 0.4 mL and *V_comp_* = 0.3 mL, respectively. At the highest compression (i.e., *V_air_* = 0.3 mL), it was possible to compress the air cavity about 0.3 mL, because a syringe needle additionally included about 0.06 mL cavity. As shown in Figure 2B, temporal variations of Δ*<I_PC_>* were obtained with respect to the air cavity (*V_air_*). Figure 2C represented microscopic images captured at a specific time when Δ*<I_PC_>* had a maximum value. As a result, when the air cavity decreased, Δ*<I_PC_>* increased significantly. Using the formula of pressure index (*PI*), variations of *PI* were obtained with respect to *V_air_*. As shown in Figure 2D, *PI* decreased by increasing *V_air_*. From the statistical test (ANOVA), PI tended to decrease significantly with respect to the air cavity (i.e., *p*-value = 0.001). In other words, because a lower value of the air cavity corresponds to a higher compression ratio (*CR*) (i.e., *CR* = *V_comp_*/*V_air_*), it contributed to increasing pressure inside ACS. According to previous studies [36,57], the image intensity of RBCs collected in a counter-fluid channel (i.e., PC named in this study) was proportional to pressure at a specific location (i.e., BC named in this study) in the microfluidic device. Therefore, highly increased pressure inside ACS causes an increase in pressure in the microfluidic channel. The results indicated that the air cavity adjusted inside ACS has the ability to control pressure in the microfluidic device. In this study, for consistent measurement of the pressure index (PI), the specification of the ACS was fixed as an air cavity volume of *V_air_* = 0.4 mL, blood volume of *V_blood_* = 0.4 mL, and air compression volume of *V_comp_* = 0.3 mL.

### 3.2. Pressure Index Variations for Blood Samples Composed of GA-Stimulated Hardened RBCs

On the basis of specification of the ACS, variations of pressure index (*PI*) were obtained by adjusting the degree of RBC deformability. RBCs aggregation was varied depending on changes of several factors, including plasma proteins, RBC membrane viscoelasticity, and hematocrit. To reduce the degree of RBCs aggregation, normal RBCs were fixed by dipping it into GA solution. Furthermore, PBS solution, which excluded plasma proteins, was used as a suspension solution. Finally, the hardened blood sample was prepared by adding hardened RBCs into PBS solution. According to previous work [51], three parameters (i.e., *Slope*, *A_ratio_*, and *A_upp_*) suggested to quantify RBCs aggregation remained constant without respect to the concentration of GA solution. The previous result indicated that hardened RBCs in PBS suspension did not contribute to varying RBCs aggregation. According to previous studies [57,62], RBCs aggregation resulted in deteriorating the measurement accuracy of pressure. Thus, to exclude the effect of RBCs aggregation, blood (*Hct* = 50%) was then prepared by adding hardened RBCs into PBS. To decrease RBC deformability, normal RBC was dipped to a specific concentration of GA solution (i.e., *C_GA_* = 5, 10, and 15 μL/mL) for a specific duration of 10 min.

**Figure 3:**
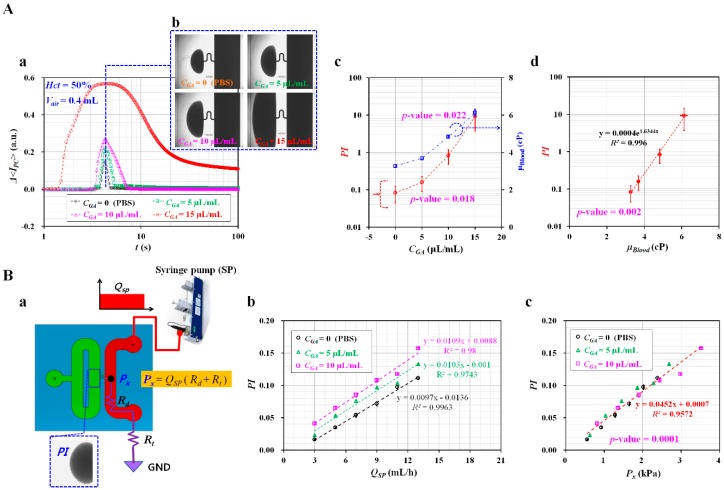
Quantitative evaluations of pressure for blood samples composed of glutaraldehyde (GA)-induced hardened RBCs. Here, the blood sample (haematocrit (*Hct*) = 50%) was consistently supplied into a microfluidic channel by operating the ACS at the same protocol (i.e., air suction = 0.4 mL, blood suction = 0.4 mL, and air compression = 0.3 mL). (**A**) Pressure measurement for various blood samples composed of RBCs hardened with various concentrations of GA solution. (**a**) Temporal variations of Δ*<I_PC_>* with respect to various concentrations of GA solution (*C_GA_* = 0, 5, 10, and 15 μL/mL). (**b**) Microscopic images captured at the time when Δ*<I_PC_>* had the highest value depending on *C_GA_*. (**c**) Variations of pressure index (*PI*) and blood viscosity (*μ_Blood_*) with respect to *C_GA_*. (**d**) Relationship between *PI* and *μ_Blood_*. (**B**) Relationship between *PI* and pressure at junction (*P_x_*) in microfluidic channel. (**a**) Schematic diagram of experimental setup. GND (i.e., Ground) means zero value of gauge pressure (i.e., *P* = 0). (**b**) Variations of *PI* with respect to *Q_SP_* and *C_GA_*. (**c**) Linear relationship between *PI* and *P_x_*.

As shown in Figure 3(A-a), variations of Δ*<I_PC_>* were obtained by varying concentrations of GA solution (*C_GA_*). In addition, Figure 3(A-b) represented microscopic images of blood captured at a specific time when Δ*<I_PC_>* had maximum value with respect to *C_GA_* = 0, 5, 10, and 15 μL/mL. Here, *C_GA_* = 0 denotes 1x PBS solution. Using temporal variations of Δ*<I_PC_>*, variations of PI were obtained with respect to *C_GA_*. As shown in Figure 3(A-c), statistical analysis (ANOVA) indicated that *PI* increased significantly by increasing *C_GA_* (i.e., *p*-value = 0.018). To compare with the corresponding PI obtained for the same blood samples, variations of blood viscosity (*μ_Blood_*) were obtained using the co-flowing method [56]. As shown in Figure S1A (Supplementary Materials), the PBS solution and blood sample were simultaneously supplied into a microfluidic device, especially at the same flow rate (i.e., *Q_PBS_* = *Q_Blood_* = *Q*, *Q_PBS_*: flow rate of PBS solution, *Q_Blood_*: flow rate of blood sample). To measure variations of blood viscosity with respect to shear rate, the specific flow rate of each fluid ranging from *Q* = 0.1 mL/h to *Q* = 3.1 mL/h at an interval of 0.2 mL/h was controlled with two syringe pumps. After setting the corresponding flow rate, microscopic images were captured at an interval of 1 s for 2 min (i.e., total number of images = 120). By conducting digital image processing, temporal variations of blood-filled width (α_Blood_) were obtained with respect to *Q*. Figure S1B (Supplementary Materials) showed temporal variations of *α_Blood_* and *Q*. As a result, α_Blood_ increased at higher concentrations of GA solution. By referring to the co-flowing method discussed in a previous study [56], variations of blood viscosity (*μ_Blood_*) were obtained with respect to shear rate ($\dot{\gamma }$). As shown in Figure S1C (Supplementary Materials), at sufficient higher shear rates ($i.e.,\;\dot{\gamma }>200\;s^{-1}$), the blood sample behaved as Newtonian fluid. In other words, blood viscosity was kept constant without respect to shear rate. When blood was supplied into a microfluidic channel from ACS, the shear rate in BC was estimated at a higher value of $\dot{\gamma }>10^{3}\;s^{-1}$, especially at an initial time (*t* < 10 s). Thus, using blood viscosity obtained at the higher shear rates, blood viscosity was quantified as mean ± standard deviation. As shown in Figure S1D (Supplementary Materials) and Figure 3(A-c), blood viscosity increased at higher concentrations of GA solution. From the statistical analysis (ANOVA), GA solution contributed significantly to increasing blood viscosity (i.e., *p*-value = 0.022). As shown in Figure 3(A-d), to find out relationship between *PI* and *μ_Blood_*, non-linear regression analysis as power-law model was conducted using the EXCEL program (Microsoft^TM^, Redmond, WA, USA). A regression analysis indicated that relationship between *PI* and *μ_Blood_* was approximately described as *PI* = 0.004 *exp* (1.6344*μ_Blood_*) (i.e., *R*^2^ = 0.996, and *p*-value = 0.002). In addition, *PI* varied largely with respect to specific concentrations of GA solution. Therefore, instead of blood viscosity (*μ_Blood_*), pressure index (*PI*) can be effectively used to monitor characteristics of blood samples (i.e., RBC deformability) from a biophysical point of view.

To find out the pressure corresponding to the pressure index (*PI*), the blood flow rate of the syringe pump (i.e., *Q_sp_*) was kept constant, as shown in Figure 3(B-a). From the simple circuit model for the microfluidic device, the pressure at junction (i.e., *P_x_*) was derived as *P_x_* = *Q_sp_* × (*R_d_* + *R_t_*). Here, *R_d_* and *R_t_* mean fluidic resistances of the microfluidic channel (i.e., BC) and tube connected to the outlet, respectively. Here, to change pressure inside the microfluidic channel, the syringe pump was set to various flow rates ranging from *Q_sp_* = 3 mL/h to *Q_sp_* = 13 mL/h, at intervals of 2 mL/h. In addition, blood samples were prepared by adding hardened RBCs into PBS solution. As shown in Figure 3(B-b), the relationship between *Q_sp_* and *PI* was evaluated by constructing an X–Y plot, and conducting linear regression analysis. From the results, R^2^ of linear regression formula was given as higher values of *R*^2^ = 0.9743~0.9963. PI increased linearly depending on flow rate. As shown in Figure 3(B-c), the X–Y plot was constructed to find out the pressure corresponding to the pressure index (*PI*). Linear regression analysis indicated that R^2^ of linear regression formula was given as a higher value of *R*^2^ = 0.9572. PI varied linearly with respect to *P_x_*. The linear regression formula was obtained as *PI* = 0. 0452 *P_x_* + 0.0007 (kPa). Minimum value of pressure was measured as *P_x_* = 0.55 kPa for *PI* = 0.016. From the results, the pressure index (*PI*) can be effectively used to monitor variations of pressure in the microfluidic channel (i.e., BC).

### 3.3. Effect of Hematocrit and Base Solutions on Pressure and RBCs aggregation Indices

Because pressure index and RBC aggregation can be substantially varied by hematocrit and base solution, it was required to evaluate the effect of contribution factors, including hematocrit and base solutions. The suggested method was employed to find out the effect of hematocrit and base solution on the measurement of pressure and RBC aggregation. Hematocrits of blood samples were adjusted to *Hct* = 30%, 40%, and 50% by adding normal RBCs into base solution (i.e., PBS solution, plasma, and dextran solution [i.e., *C_dextran_* = 10 mg/mL]). 

**Figure 4:**
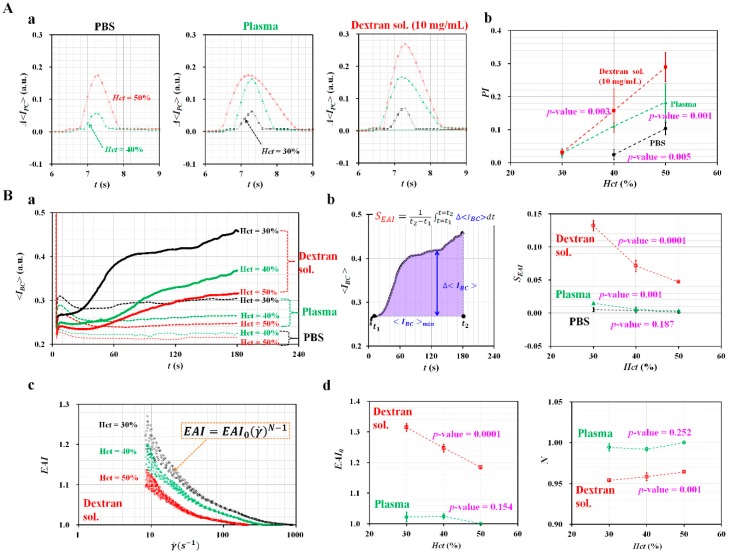
Quantitative evaluation of the effect of hematocrit and base solution on pressure and RBC aggregation. (**A**) Variations of pressure (*PI*) with respect to hematocrit (*Hct*) (*Hct* = 30%, 40%, and 50%) and base solution (PBS, plasma, and dextran solution (10 mg/mL)). (**a**) Temporal variations of Δ*<I_PC_>* with respect to hematocrit and base solution. (**b**) Variations of *PI* with respect to hematocrit and base solution. (**B**) Variations of RBC aggregation (*EAI*) with respect to hematocrit and base solution. (**a**) Temporal variations of *<I_BC_>* with respect to hematocrit and base solution. (**b**) Variations of *S_EAI_* with respect to hematocrit and base solution. (**c**) Variations of *EAI* with respect to shear rate ($\dot{\gamma }$). (**d**) Variations of *EAI*_0_ and *N* with respect to hematocrit and base solution.

First, the contribution of *Hct* and base solution on pressure index (*PI*) was evaluated quantitatively. As shown in Figure 4(A-a), temporal variations of Δ*<I_PC_>* were obtained by varying hematocrit and base solution. With respect to the blood sample (*Hct* = 30%, normal RBCs suspended in PBS solution), blood did not move into PC from BC. As there was no RBCs collected in PC, Δ*<I_PC_>* remained zero. However, the other eight blood samples showed significant variations of Δ*<I_PC_>* because they moved into PC and returned to BC. As a result, the Δ*<I_PC_>* increased considerably at a higher value of hematocrit. At the same hematocrit, the dextran solution contributed to increasing Δ*<I_PC_>* significantly, when compared with different base solutions (i.e., PBS solution and plasma). As shown in Figure 4(A-b), variations of *PI* were obtained by varying hematocrit and base solution. From the statistical analysis (ANOVA), the *PI* varied significantly with respect to hematocrit (i.e., *p*-value = 0.005 for PBS solution, *p*-value = 0.001 for plasma, and *p*-value = 0.003 for dextran solution). The results indicated that the *PI* can be used effectively to monitor variation of hematocrit and base solution.

Second, the effects of hematocrit and base solution on RBC aggregation were evaluated quantitatively. As shown in Figure 4(B-a), temporal variations of *<I_BC_>* were obtained by varying hematocrit and base solution. As shown in Figure 1(C-b), the blood flow rate (*Q_μPIV_*) decreased over time because the air cavity inside ACS increased continuously. After a certain amount of time had elapsed, the blood flow rate decreased to the corresponding shear rate where RBC aggregation occurred. Then, the *<I_BC_>* increased over time because RBCs were aggregated continuously. When compared with PBS solution, dextran solution and plasma contributed significantly to increasing *<I_BC_>*, owing to the effect of RBCs aggregation. In addition, a lower value of hematocrit exhibited a significant increase in *<I_BC_>*, which indicated that RBCs aggregation tended to increase at lower values of hematocrit. To quantify RBCs aggregation without information on shear rate, the RBC aggregation index (*S_EAI_*) was newly suggested. As shown in the left panel of Figure 4(B-b), S_EAI_ was calculated by averaging Δ*<I_BC_>* from *t* = *t*_1_ to *t* = *t*_2_. Here, Δ*<I_BC_>* is calculated as Δ*<I_BC_>* = *<I_BC_>* − *<I_BC_>_min_*. The right panel of Figure 4(B-b) showed variations of *S_EAI_* with respect to hematocrit and base solution. From the statistical analysis (ANOVA), two base solutions (plasma and dextran solution) exhibited significant variations of *S_EAI_* with respect to hematocrit (i.e., *p*-value = 0.001 for plasma, and *p*-value = 0.0001 for dextran solution), when compared with PBS solution (i.e., *p*-value = 0.187). The results indicated that the *S_EAI_* can be effectively used to monitor variations of RBCs aggregation when information on the shear rate is not available. On the other hand, temporal variations of blood flow rate (*Q_μPIV_*) were obtained by conducting time-resolved micro-PIV. The corresponding shear rate ($\dot{\gamma }$) was then estimated by inserting *Q_μPIV_* into the formula of shear rate for a cross-sectional microfluidic channel (i.e., $\dot{\gamma }=\frac{6Q_{\mu PIV}}{w\;h^{2}}$) [56]. In this study, when *Q_μPIV_* was less than 0.1 mL/h, the shear rate did not provide a consistent value because a micro-PIV technique showed a technical limitation on the measurement of extremely lower blood flow rate. For this reason, the shear rate was estimated up to a blood flow rate of 0.1 mL/h (i.e., lower threshold of shear rate = 8.3 s^−1^). As shown in Figure 4(B-c), with respect to the blood sample (*Hct* = 30%, 40%, and 50%) suspended in dextran solution, RBCs aggregation index (*EAI*) was obtained by varying the shear rate. As a result, lower hematocrit (*Hct* = 30%) showed higher value of *EAI*, when compared with higher hematocrit (*Hct* = 50%). The power-law model as a non-linear regression formula was suggested as $EAI=EAI_{0}{\left(\dot{\gamma }\right)}^{N-1}$ to quantify *EAI* with respect to the shear rate. Two unknown parameters (*N*, and *EAI*_0_) were obtained by conducting non-linear regression analysis with the EXCEL program (Microsoft^TM^, Redmond, WA, USA). As shown in Figure 4(B-d), variations of *N* and *EAI*_0_ were obtained by varying hematocrit and base solution. From the statistical analysis (ANOVA), dextran solution (i.e., *p*-value = 0.0001) contributed to decreasing *EAI*_0_ significantly with respect to hematocrit, when compared with plasma (i.e., *p*-value = 0.154). In addition, dextran solution (i.e., *p*-value = 0.001) contributed to increasing *N* significantly with respect to hematocrit, when compared with plasma (i.e., *p*-value = 0.252). When compared with *S_EAI_*, *EAI* was estimated using variations of *<I_BC_>* obtained above $\dot{\gamma }=8.3{\;s}^{-1}$. Thus, S_EAI_ (i.e., *p*-value < 0.05) was more effective than EAI (i.e., *p*-value > 0.05) for quantifying RBC aggregation of the blood sample (i.e., normal RBCs in plasma suspension). From the experimental results, two indices (*S_EAI_*, and *EAI*) can be effectively employed to evaluate the variations of RBC aggregation, without and with information on the shear rate. 

### 3.4. Quantification of RBC Aggregation-Enhanced Blood Samples

**Figure 5:**
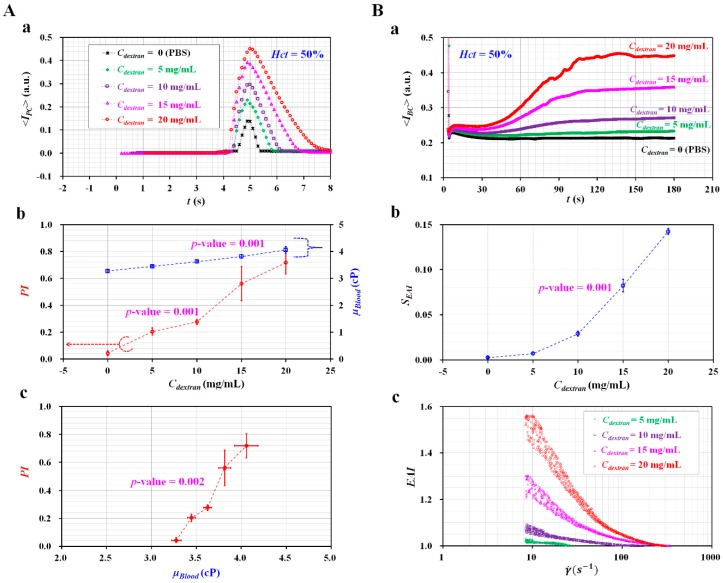
Performance evaluation of the proposed method for various blood samples. To enhance RBCs aggregation, hematocrit of blood sample (*Hct* = 50%) was prepared by adding normal RBCs into specific concentrations of dextran solution (*C_dextran_* = 0, 5, 10, 15, and 20 mg/mL). (**A**) Variations of pressure index (*PI*) with respect to concentration of dextran solution (*C_dextran_*). (**a**) Temporal variations of Δ*<I_PC_>* with respect to *C_dextran_*. (**b**) Variations of *PI* and *μ_Blood_* with respect to *C_dextran_*. (**c**) Relationship between *PI* and *μ_Blood_*. Regression analysis exhibits a significant relationship between *PI* and *μ_Blood_* (i.e., *R*^2^ = 0.9757 and *p*-value = 0.002). (**B**) Quantification of two RBCs aggregation indices (*S_EAI_*, *EAI*) with respect to *C_dextran_*. (**a**) Temporal variations of *<I_BC_>* with respect to *C_dextran_*. (**b**) Variations of *S_EAI_* with respect to *C_dextran_*. From the statistical analysis (analysis of variance (ANOVA)), *S_EAI_* increases significantly with respect to *C_dextran_* (*p*-value = 0.001). (**c**) Variations of *EAI* with respect to the shear rate ($\dot{\gamma }$).

Finally, the performance of the proposed method was evaluated by measuring blood samples with different degrees of RBCs aggregation. A dextran solution was generally employed to substantially increase RBCs aggregation. To simulate RBC aggregation considerably, various blood samples (*Hct* = 50%) were prepared by adding normal RBCs into specific concentrations of dextran solution (i.e., *C_dextran_* = 0, 5, 10, 15, and 20 mg/mL). As shown in Figure 5(A-a), temporal variations of Δ*<I_PC_>* were obtained by varying concentrations of dextran solution. As a result, the Δ*<I_PC_>* tended to increase significantly, at higher concentrations of dextran solution. As shown in Figure 5(A-b), variations of *PI* were obtained with respect to *C_dextran_*. To compare with PI, the co-flowing method [56] was employed to measure blood viscosity (*μ_Blood_*) for the same blood samples. From the statistical analysis (ANOVA), both properties (*PI* and *μ_Blood_*) were varied significantly with respect to *C_dextran_* (i.e., *p*-value = 0.001 for *PI*, and *p*-value = 0.001 for *μ_Blood_*). 

As shown in Figure 5(A-c), a linear regression was conducted to find out the relationship between *PI* and *μ_Blood_*. A regression analysis exhibited a significant relationship between *PI* and *μ_Blood_* (i.e., *R*^2^ = 0.9757, and *p*-value = 0.002). In other words, the *PI* exhibited significant variations with respect to *C_dextran_*, when compared with blood viscosity. From the results, the *PI* can be effectively used to monitor variations of blood samples as an alternative to blood viscosity. When measuring blood viscosity for monitoring variations of blood samples (i.e., hematocrit or base solution), two syringe pumps were required to control flow rates of both fluids precisely [66,67,68,69]. However, two syringe pumps can be removed by adopting the pressure index (*PI*) instead of blood viscosity (*μ_Blood_*). 

To quantify variations of RBCs aggregation with respect to *C_dextran_*, variations of *<I_BC_>* were obtained over time. As shown in Figure 5(B-a), the *<I_BC_>* tended to increase significantly at higher concentrations of dextran solution. Without information on the shear rate, variations of RBCs aggregation index (*S_EAI_*) were obtained by averaging *<I_BC_>* for specific intervals of time. As shown in Figure 5(B-b), variations of *S_EAI_* were obtained by varying *C_dextran_*. According to the statistical analysis (ANOVA), *S_EAI_* varied significantly (i.e., *p*-value = 0.001). On the other hand, with information on the shear rate, variations of the RBC aggregation index (*EAI*) were obtained by varying the shear rate. 

To validate the blood flow rate obtained by conducting the micro-PIV technique, the blood flow rate obtained with the micro-PIV technique (*Q_μPIV_*) was compared with a given flow rate controlled by a syringe pump (*Q_sp_*). Using a syringe pump, the flow rate of blood (*Q_sp_*) increased sequentially from *Q_sp_* = 0.1 mL/h to *Q_sp_* = 1.5 mL/h, at an interval of 0.2 mL/h. Each flow rate was maintained for 2 min. Figure S2A (Supplementary Materials) showed temporal variations of *Q_sp_* and *Q_μPIV_* with respect to *C_dextran_*. As shown in Figure S2B (Supplementary Materials), an X–Y plot was constructed to find out the linear relationship between *Q_sp_* and *Q_μPIV_*. From the regression analysis, R^2^ of the linear regression formula exhibited a higher value (i.e., *R*^2^ = 0.99). The result indicated that both flow rates have a significantly linear relationship. As shown in Figure S2C (Supplementary Materials), the slope of linear regression (i.e., Δ*Q_μPIV_*/Δ*Q_sp_*) varied from 0.9158 to 1.0153 with respect to *C_dextran_*. From the results, it led to the conclusion that the flow rate obtained by conducting the micro-PIV technique (*Q_μPIV_*) showed a maximum measurement error of 8.5%, when compared with *Q_sp_* controlled by a syringe pump (i.e., reference flow rate). As shown in Figure 5(B-c), a higher concentration of dextran solution contributed to increasing *EAI* significantly, when compared with a lower concentration of dextran solution. On the basis of the power-law model (i.e., $EAI=EAI_{0}{\left(\dot{\gamma }\right)}^{N-1}$), two unknown parameters (*EAI*_0_ and *N*) were obtained by conducting a non-linear regression analysis. 

**Figure 6:**
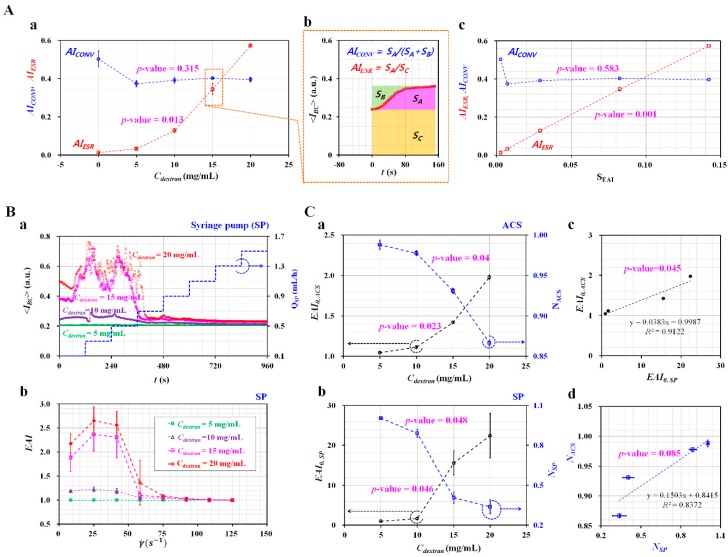
Quantitative comparison of RBCs aggregation between the proposed method and the previous method. Blood sample (*Hct* = 50%) was prepared by adding normal RBCs into a specific dextran solution (*C_dextran_* = 0, 5, 10, 15, and 20 mg/mL). (**A**) Comparison between *S_EAI_* (i.e., without information on shear rate) and previous RBCs aggregation indices (*AI_conv_*, *AI_ESR_*). (**a**) Variations of *AI_conv_* and *AI_ESR_* with respect to *C_dextran_*. (**b**) Definition of the previous RBCs aggregation indices: *AI_conv_* = *S_A_*/(*S_A_* + *S_B_*), and *AI_ESR_* = *S_A_*/*S_C_*. (**c**) The relationship between proposed RBCs aggregation index (*S_EAI_*) and previous RBCs aggregation indices (*AI_conv_*, *AI_ESR_*). (**B**) Quantitative evaluation of *EAI* (i.e., with information on shear rate) with respect to ACS and syringe pump. (**a**) Temporal variations of *EAI* obtained using the syringe pump. (**b**) Temporal variations of EAI obtained by *ACS*. (**C**) Comparison of two parameters (*EAI*_0_, and *N*) obtained by ACS and the syringe pump (SP). (**a**) Variations of *EAI*_0*,ACS*_ and *N_ACS_* obtained by ACS with respect to *C_dextran_*. (**b**) Variations of *EAI*_0*,SP*_ and *N_SP_* obtained using the syringe pump with respect to *C_dextran_*. (**c**) Relationship between *EAI*_0*,ACS*_ and *EAI*_0*,SP*_. (**d**) Relationship between *N_ACS_* and *N_ACS_*.

As illustrated in Figure 6(A-b), two indices (i.e., *AI_CONV_* [54] and *AI_ESR_* [52,55,57]) as previous methods were employed to evaluate the variations of *S_EAI_* with the concentration of dextran solution. Figure 6(A-a) showed variations of *AI_CONV_* and *AI_ESR_* with respect to *C_dextran_*. According to the experimental results, *AI_ESR_* was varied significantly at higher concentrations of dextran solution (i.e., *p*-value = 0.013). However, *AI_conv_* remained constant without respect to *C_dextran_* (i.e., *p*-value = 0.315). Because dextran contributed to increasing RBCs aggregation substantially [52], the *AI_ESR_* could be considered as effective when compared with the *AI_conv_*. Furthermore, as shown in Figure 6(A-c), the linear relationship between the proposed index (*S_EAI_*) and previous indices (*AI_conv_*, and *AI_ESR_*) was verified by drawing an X–Y plot and conducting a regression analysis. As a result, the *S_EAI_* represented variations in *AI_ESR_* appropriately (i.e., *p*-value = 0.001). However, *S_EAI_* was independent of *AI_conv_* (i.e., *p*-value = 0.583). According to previous studies, *AI_ESR_* as a dimensionless index was suggested to include the effect of ESR (erythrocyte sedimentation rate) in the syringe for a long elapsed time (~2100 s) [55]. In this study, because the experiment was completed within a short time (~200 s), ESR in the ACS was considered as negligible. In other words, ESR did not contributed to varying RBCs aggregation. For this reason, the *AI_ESR_* and *S_EAI_* showed the same meaning, and a similar trend of RBCs aggregation. 

To compare *EAI* (i.e., with information on shear rate) obtained by ACS, the ACS used for delivering blood was replaced with the syringe pump. By controlling the syringe pump, the blood flow rate increased from 0.1 mL/h to 1.3 mL/h at an interval of 0.2 mL/h, in a stepwise fashion. As shown in Figure 6(B-a), temporal variations of image intensity of blood (*<I_BC_>*) were obtained with respect to *C_dextran_*. On the basis of the definition of *EAI* suggested in this study, the variations of *EAI* were obtained with respect to the shear rate and *C_dextran_*. As shown in Figure 6(B-b), dextran solution contributed substantially to increasing *EAI*. In addition, *EAI* decreased substantially at higher shear rates (i.e., $\dot{\gamma }>108.3{\;s}^{-1}$). To evaluate the difference between *EAI* obtained from the ACS and *EAI* obtained from the syringe pump, the unknown two parameters in the power-law model (i.e., *EAI* = $EAI_{0}{\left(\dot{\gamma }\right)}^{N-1}$) were obtained by conducting non-linear regression analysis. Figure 6(C-a) showed variations of *EAI*_0, *ACS*_ and *N_ACS_* obtained by the ACS with respect to *C_dextran_*. Two parameters varied significantly with respect to C_dextran_ (i.e., *p*-value = 0.023 for *EAI*_0,*ACS*_, and *p*-value = 0.04 for *N_ACS_*). On the contrary, Figure 6(C-b) exhibited variations of *EAI*_0,*SP*_ and *N_SP_* obtained by the syringe pump (SP) with respect to *C_dextran_*. When compared with *EAI* obtained by ACS, the statistical analysis (ANOVA) indicated that the two parameters were changed substantially with respect to *C_dextran_* (i.e., *p*-value = 0.046 for *EAI*_0_, and *p*-value = 0.048 for *N*). A regression analysis was conducted to examine the relationship between *EAI* obtained by the ACS and *EAI* obtained by the SP. As shown in Figure 6(C-c,C-d), the regression analysis indicated that SP and ACS exhibited a significant linear relationship (i.e., *p*-value = 0.045 for *EAI*_0_, and *p*-value = 0.085 for *N*). From the results, *EAI* obtained by ACS provided consistent values when compared with one obtained by the syringe pump.

**Figure 7:**
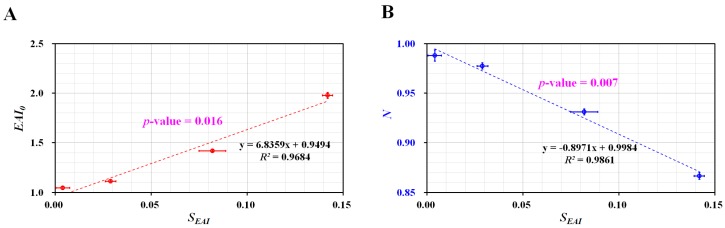
Quantitative evaluation of relationship between *S_EAI_* and *EAI* (*EAI*_0_, *N*). (**A**) Evaluation of relationship between *EAI*_0_ and *S_EAI_*. *EAI*_0_ and *S_EAI_* exhibit a significant linear relationship (i.e., *R*^2^ = 0.9684, *p*-value = 0.016). (**B**) Evaluation of relationship between *N* and *S_EAI_.* Linear regression analysis indicates that index (*N*) decreases significantly with respect to *S_EAI_* (i.e., *R*^2^ = 0.9861, *p*-value = 0.007).

Finally, a linear regression analysis was conducted to find out the relationship between *S_EAI_* (i.e., without information on shear rate) and EAI (*EAI*_0_, and *N*) (i.e., with information on shear rate). As shown in Figure 7A, the relationship between *EAI*_0_ and *S_EAI_* was represented with an X–Y plot. The regression analysis indicates that *EAI*_0_ and *S_EAI_* exhibit a significant linear relationship (i.e., *R*^2^ = 0.9684, and *p*-value = 0.016). As shown in Figure 7B, a relationship between *N* and *S_EAI_* was represented with an X–Y plot. The regression analysis indicates that index (*N*) decreases significantly with respect to *S_EAI_* (i.e., *R*^2^ = 0.9861, *p*-value = 0.007). 

In this study, while adopting the ACS (air-compressed syringe), the blood sample was successfully supplied into a microfluidic device without a bulk-sized syringe pump. However, quantification of the blood flows was available by analyzing microscopic images obtained from an optical microscope and high-speed camera. As a limitation, this proposed method was still demonstrated in well-equipped laboratory. To resolve the issue, the proposed method should be improved by adopting a portable image acquisition system [70]. In the near future, it will be then employed to measure multiple hemorheological properties including pressure, RBC deformability, RBC aggregation, and hematocrit. 

## 4. Conclusions

In this study, a simple method for sequential measurement of pressure and RBC aggregation was proposed by quantifying blood flow (i.e., velocity and image intensity) through a microfluidic device, in which an air-compressed syringe (ACS) is used to control sample injection. Pressure was quantified using the pressure index (*PI*) at an initial time (*t* < 10 s). PI depends significantly on the characteristics of the samples used (i.e., hematocrit or base solution), when compared with blood viscosity. After a certain of time has elapsed (*t* > 30 s), RBC aggregation was quantified using S_EAI_ and *EAI*. *S_EAI_* (i.e., without information on shear rate) and *EAI* (i.e., with information on shear rate) were found to vary significantly depending on the degree of RBC aggregation. Using the ACS (i.e., air suction = 0.4 mL, blood suction = 0.4 mL, and air compression = 0.3 mL), this proposed method could provide information on pressure and RBC aggregation in a 3 min experiment. This study included substantial differences when compared with previous works [51,52,62,63]. First, two RBC aggregation indices (*S_EAI_*, and *EAI*) were suggested and quantified under blood delivery of blood by employing an ACS. Second, owing to the ACS, the blood flow rate in a microfluidic channel continuously decreased over time. It contributed to removing on–off flow control, which was considered a necessary step in the previous studies. Finally, the pressure index (*PI*) was employed to monitor contribution of hematocrit as an alternative to blood viscosity. In conclusion, the proposed method is capable of measuring RBCs aggregation consistently using a microfluidic device, even without the need for external bulky delivery of blood samples.

## Figures and Tables

**Table 1:** Several issues raised in the previous measurement method of red blood cells’ (RBCs) aggregation under microfluidic environments. ESR, erythrocyte sedimentation rate.

Blood Biophysical Properties	Blood Delivery Tools	Issues	References
RBCs aggregation, and blood pressure	Disposable air-compressed pump (on–off flow control)	- RBCs aggregation was quantified by stopping blood flows with pinch valve (i.e., on–off flow control) - RBCs aggregation was quantified at stasis	[62]
RBCs aggregation, RBC deformability, and hematocrit	Syringe pump (periodic on–off control)	- Bulk-sized syringe pump was employed to turn on and off blood flows periodically	[63]
RBCs aggregation, and blood viscosity	Two syringe pumps (constant flow rate)	- Two syringe pumps were used to deliver blood flows continuously	[52]
RBCs aggregation, and ESR	Air suction syringe, or syringe pump	- It was impossible to monitor the effect of hematocrit or base solution	[18,55,57]

## Supplementary Materials

The following are available online at https://www.mdpi.com/2072-666X/10/9/577/s1, Figure S1: quantification of blood viscosity composed of RBCs hardened with GA solution, and Figure S2: validation of blood flow rate obtained by conducting micro-PIV.
